# Lake eutrophication prediction based on improved MIMO-DD-3Q Learning

**DOI:** 10.1371/journal.pone.0294278

**Published:** 2023-11-14

**Authors:** Li Wang, Chaoran Ning, Xiaoyi Wang, Jiping Xu, Zhiyao Zhao, Jiabin Yu, Huiyan Zhang, Qian Sun, Yuting Bai, Xuebo Jin, Qianhui Tang

**Affiliations:** 1 Beijing Laboratory for Intelligent Environmental Protection, School of Artificial Intelligence, Beijing Technology and Business University, Beijing, China; 2 Beijing Institute of Fashion Technology, Beijing, China; King Fahd University of Petroleum and Minerals, SAUDI ARABIA

## Abstract

As for the problem that the traditional single depth prediction model has poor strain capacity to the prediction results of time series data when predicting lake eutrophication, this study takes the multi-factor water quality data affecting lake eutrophication as the main research object. A deep reinforcement learning model is proposed, which can realize the mutual conversion of water quality data prediction models at different times, select the optimal prediction strategy of lake eutrophication at the current time according to its own continuous learning, and improve the reinforcement learning algorithm. Firstly, the greedy factor, the fixed parameter of Agent learning training in reinforcement learning, is introduced into an arctangent function and the mean value reward factor is defined. On this basis, three Q estimates are introduced, and the weight parameters are obtained by calculating the realistic value of Q, taking the average value and the minimum value to update the final Q table, so as to get an Improved MIMO-DD-3Q Learning model. The preliminary prediction results of lake eutrophication are obtained, and the errors obtained are used as the secondary input to continue updating the Q table to build the final Improved MIMO-DD-3Q Learning model, so as to achieve the final prediction of water eutrophication. In this study, multi-factor water quality data of Yongding River in Beijing were selected from 0:00 on July 26, 2021 to 0:00 on September 5, 2021. Firstly, data smoothing and principal component analysis were carried out to confirm that there was a certain correlation between all factors in the occurrence of lake eutrophication. Then, the Improved MIMO-DD-3Q Learning prediction model was used for experimental verification. The results show that the Improved MIMO-DD-3Q Learning model has a good effect in the field of lake eutrophication prediction.

## 1 Introduction

Rivers and lakes are very important fresh water resources in China, and also one of the precious resources that people depend on for survival. Recently, with the rapid development of our social economy and the improvement of human activities, lake eutrophication [1] has become the primary problem of river and lake treatment in China. The occurrence of lake eutrophication [2] is jointly affected by several indexes, such as physical and chemical indexes, biochemical indexes and nutrient salt indexes [3]. These include:KMNO_4_, COD,BOD_5_,TOC, NH_3_-N, chroma, conductivity, TDS, turbidity, NO_3_-N, Chl-a [4] and fluoride. The increase or decrease of these factors will have a certain impact on the eutrophication of lake [5], and then affect the water ecological balance of the whole river and lake. In recent years, lake eutrophication in different degrees has occurred in many rivers and lakes in China, which has also caused some harm. In the past decade, for example, there have been multiple bloom outbreaks in Lake Wu [6], which led to the sudden drop of dissolved oxygen in the water and the death of a large number of fish, resulting in serious lake eutrophication problems [7]. From 2016 to 2018, Chaohu Lake was evaluated according to the TLI method, and some waters in Chaohu Lake showed mild and moderate eutrophication, so it is a necessary research direction to predict lake eutrophication [8].

At present, the prediction modeling methods [9] of lake eutrophication are mainly divided into two categories: the mechanism-driven prediction modeling method [10] of lake eutrophication and the data-driven prediction modeling method [11]. The modeling methods of lake eutrophication driven by mechanism can be divided into three categories: firstly, the single nutrient load model which only considers the limiting factors is generated, and this kind of model has a vague expression of lake eutrophication [12] and has great limitations. Secondly, the multi-nutrient load model appeared, which was not suitable for rivers and lakes with a large spatial geographic range and was affected by spatial geographic location and region. Finally, it is a complex dynamic model [13] based on the combination of hydrodynamics [14] and ecosystem changes, which reflects the growth law and characteristics of physical and chemical indexes to reflect the eutrophication of lake [15]. However, this kind of model is complicated to construct and difficult to accurately fit the actual situation. Therefore, it cannot accurately predict the eutrophication of lake only based on the mechanism.

The data-driven modeling method for lake eutrophication prediction is to analyze and mine a large number of historical monitoring data. It does not take into account the physical, chemical and biological relationships among various indicators, nor does it require prior knowledge, but only considers the internal laws hidden in the data information of the system. Therefore, it is widely used in the prediction of lake eutrophication. However, most of the current prediction methods for lake eutrophication use a single data-driven model for prediction, such as machine learning, regression model grey theory model, etc. [16–18], but these models all have problems such as low prediction accuracy or too long prediction time.

Water quality concentration data that produce lake eutrophication are characterized by multiple indexes, temporal correlation, and strong data mutancy, so deep learning algorithms that are good at data analysis are generally selected for the prediction of such data [19]. The long and short term memory network can accurately capture the internal relationship between the front and back elements in the time series data, and form short-term memory by forgetting the front elements to guide the back elements, while retaining the guidelines to form long-term memory [20, 21]. In the deep random forest, key variables are found and sorted through the input data through the multi-grain scanning process, features are captured according to the sliding window, and features are fully captured and processed data are recorded in the cascade forest process [22, 23]. Transformer is a kind of neural network with self-attention mechanism, which can use time series data as the input of encoder in Transformer model and predict future values in an autoregressive way in the decoder part [24, 25]. However, the data of water quality concentration resulting in lake eutrophication are affected by climate, temperature and other factors, and the data will produce abrupt values. Therefore, a time series modeling method suitable for multi-factor prediction of lake eutrophication was adopted in this study by combining multiple types of traditional single prediction models and applying different prediction algorithms for different periods of data.

Traditional Reinforcement Learning [26] Agent interact with the surrounding environment in an unknown environment according to the "Exploration-Utilization" code of conduct, conduct observation and analysis through continuous exploration and discovery, and then continue to learn according to the rewards and punishments obtained, and finally obtain an optimal decision-making process [27]. When traditional reinforcement learning deals with specific learning tasks, the key lies in the establishment of the Agent own state space and action space, as well as the way of interaction with the environment, so as to enable the Agent to find the optimal strategy in the specific learning task. In the field of lake eutrophication prediction, Deep Reinforcement Learning [28] makes use of its powerful computing power and deep data mining ability to observe the internal relationship between various factors. At the same time, it relies on the learning decision-making ability of Reinforcement Learning and the nature of considering long-term returns to optimize a single model so as to achieve better prediction effect [29]. Therefore, it is an urgent problem to be solved in the field of lake eutrophication prediction to build a deep reinforcement learning model [30, 31] that can contain multiple factors and clearly capture the temporal correlation between data.

Aiming at the problem that the above existing technologies are not accurate enough to deal with abrupt change data in the field of lake eutrophication prediction, this study proposes a prediction method of lake eutrophication based on the Improved MIMO-DD-3Q Learning model, which solves the problem that the prediction results of a single depth prediction model are biased when the multi-factor time series data fluctuates greatly. Meanwhile, the Reinforcement Learning algorithm is improved. The problem that the training efficiency of Reinforcement Learning Agent is slow and it is easy to fall into local optimal [32, 33] is solved. At the same time, the error correction of prediction results [34, 35] is carried out to improve the prediction accuracy of the model and provide a new way of thinking for the field of lake eutrophication prediction.

## 2 Improved MIMO-DD-3Q Learning

### 2.1 Construction deep Q Learning model

In traditional Q Learning, Agent learn and update according to the “Exploration-Utilization” code of conduct. Excessive exploration will lead to the decrease of Agent learning efficiency and slow updating of Q Learning strategy, while excessive utilization will lead to the Agent easily falling into local optimization, reducing the accuracy of Q Learning strategy, and greatly increasing the training and learning time. Aiming at this problem, the original linear behavior criterion of Agent is improved in this step. Firstly, a parameter of Q Learning, the greed factor ε, is defined, and the arctangent function is introduced. The greedy factor parameter of the Agent is changed according to a certain trend, the specific changes are as follows:

$$\varepsilon =\tan^{-1}2u-0.21\pi$$ (1)

**Fig 1:**
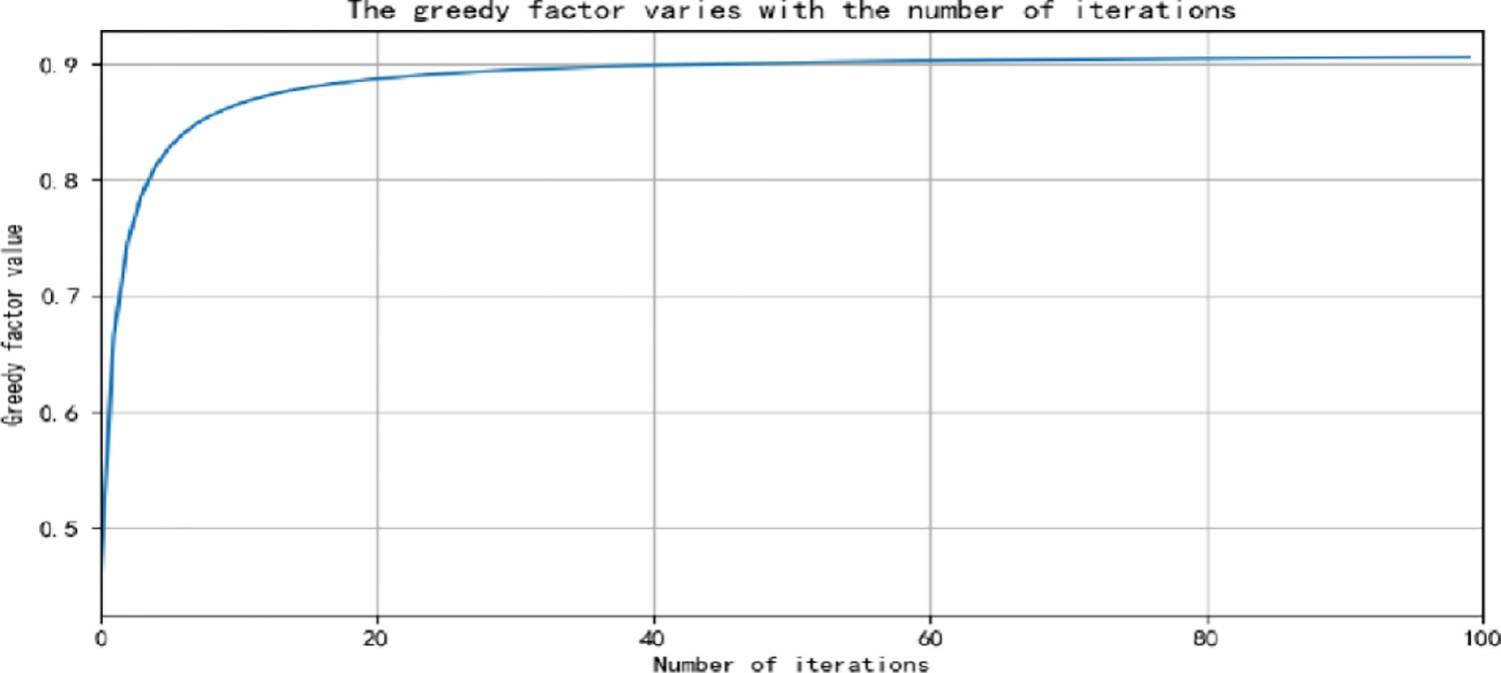
Curve of greedy factor with the number of iterations after the introduction of arctangent function.

Where, u represents the U-th training, and the curve of greed factor ε changing with the number of iterations is shown in Fig 1.

Secondly, multiple deep learning prediction models are defined as state space sets of S_*t*_, reinforcement learning training, which can be expressed as follows:

$$S_{t}=\{S_{1},\cdots ,S_{L},\cdots ,S_{W}\}$$

Where, *S*_*L*_ is the dynamic prediction model at time *t*, and *W* is the number of optional prediction models at time *t*. The action space set A_*t*_ for prediction based on the prediction model obtained from the current state is defined as reinforcement learning, which is expressed as follows:

$$A_{t}=\{A_{1},\cdots ,A_{L},\cdots ,A_{K}\}$$

Where, *A*_*L*_ is the actions predicted by the L-th prediction model at time *t*, and *K* is the number of actions that may occur after the current state is selected and predicted at time *t*.

After the above definition of the state space and action space of reinforcement learning, the agent can obtain the current prediction results of multiple indicators after single-step training. In order to enable deep Q Learning to better solve the Markov decision process, this model defines the reward factor of reinforcement learning as multi-index mean reward, which is expressed as follows:

$$R_{ave}=\frac{1}{s}\sum_{1}^{s}R_{I}$$ (2)

$$R_{I}=-|y_{t}-y_{p}|$$ (3)

Where, *s* is the number of prediction indicators, *R*_*ave*_ is the average value of reward values of multiple prediction indicators, *R*_*I*_ is the reward factor of the *I*-th prediction indicator, *y*_*t*_ is the true value of this prediction indicator, and *y*_*p*_ is the predicted value of this prediction indicator.

### 2.2 Construction MIMO deep 3Q Learning model

At time *t*, the agent interacts with the environment. According to the actions made at the current time, the average reward value *R*_*ave*_ and the state *S*′ at time *t*+1 are obtained. Furthermore, three estimated Q values are obtained according to the state *S*′ at time *t*+1, which are expressed as follows:

$$Q_{i}(S^{\prime },A^{\prime }),\;i=1,2,3$$ (4)

Where, *Q*_*i*_(*S*′, *A*′) represents the i-th estimated value of Q function selected, *A*′ is the action selected at time *t*+1, and *S*′ is the state at time *t*+1.

**Fig 2:**
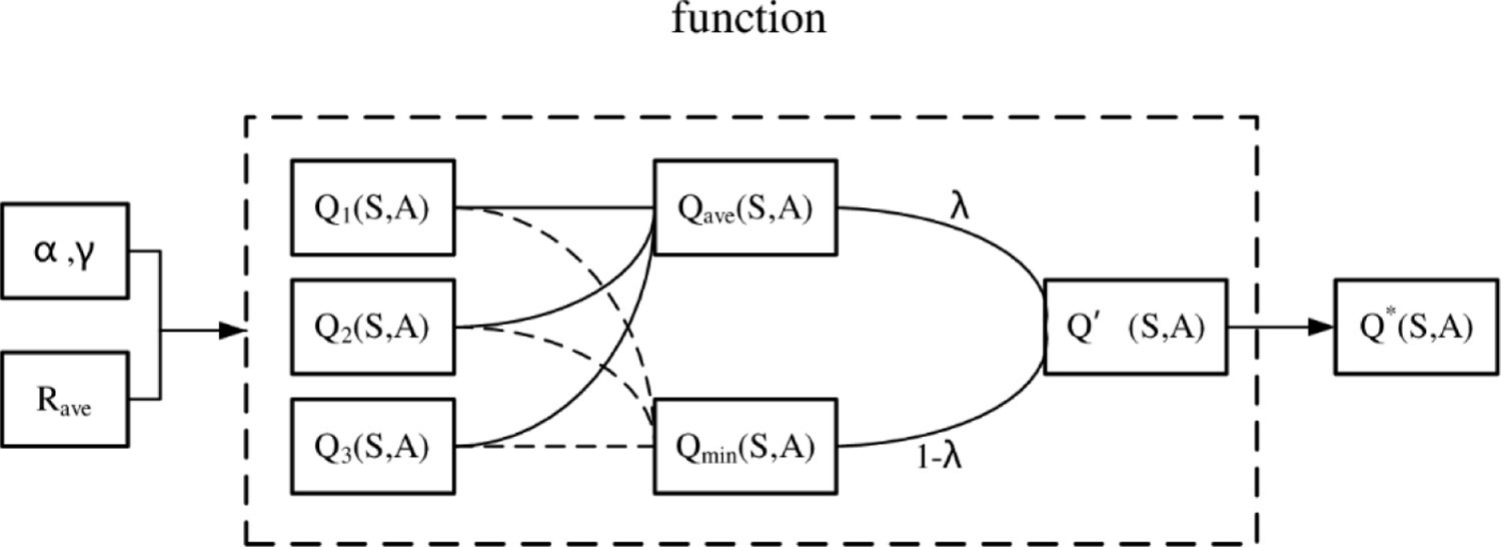
Flow chart of updating Q table after the introduction of weight parameters.

According to the three estimated Q values, the three real Q values at the previous moment are updated. Then, the three real Q values at the current moment are calculated by calculating the average value and minimum value. A constant is introduced to obtain the weight parameter with the obtained average value and minimum value, and finally the weighted Q value is calculated to obtain the final Q Learning strategy. The calculation method is shown in Fig 2.

The estimated Q value is obtained and the Q value of the previous time is updated in the following way:

$$Q_{i}(S,A)\leftarrow Q^{*}(S,A)+\alpha [R_{ave}+\gamma {max}_{A^{\prime }}Q_{i}(S^{\prime },A^{\prime })-Q^{*}(S,A)]$$ (5)

$$Q_{ave}(S,A)=\frac{1}{3}\sum_{i=1,2,3}Q_{i}(S,A)$$ (6)

$$Q_{min}(S,A)=\underset{i=1,2,3}{\min}Q_{i}(S,A)$$ (7)

$$\lambda_{\left(S,A\right)}=\frac{\left|Q_{ave}(S,A)-Q_{min}(S,A)\right|}{c+|Q_{ave}(S,A)-Q_{min}(S,A)|}$$ (8)

$$Q^{*}(S,A)=\lambda_{\left(S,A\right)}Q_{ave}(S,A)+(1-\lambda_{\left(S,A\right)})Q_{min}(S,A)$$ (9)

In the formula, *α* is the learning rate of the Agent in Q Learning, *γ* is the decay coefficient of the Agent learning in Q Learning, *c* is A constant, *Q*_*ave*_(*S*,*A*) represents the average value of the three Q values in the current state, *Q*_*min*_(*S*,*A*) represents the minimum value of the three Q values in the current state, *λ*_(*S*,*A*)_ represents the weight parameter in the current state, Q*(*S*,*A*) represents the final Q Learning strategy in the current state. In Formula (5), *max*_*A*′_*Q*_*i*_(*S*′,*A*′) said choice of the i-th Q estimate, *i* = 1,2,3.

### 2.3 Construction MIMO-DD-3Q Learning model

**Fig 3:**
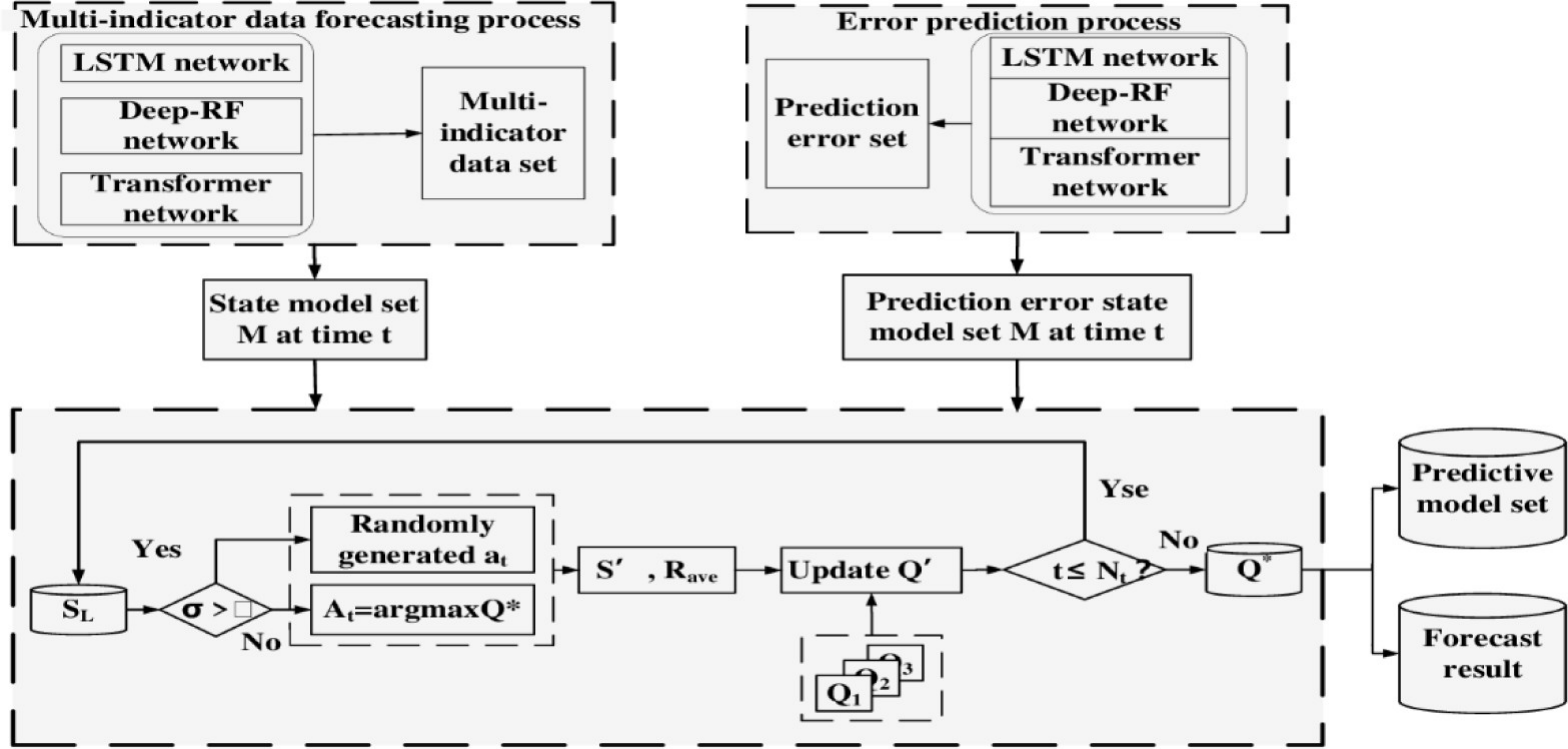
Structure diagram of Improved MIMO-DD-3Q Learning model.

The preliminary prediction results are obtained by Improving MIMO-DD-3Q Learning model. After obtaining error values according to the obtained results, multiple groups of error data are used as the second input of the Deep Q Learning model. Then, Improving MIMO-DD-3Q Learning model is constructed through the improved deep 3Q learning training, which can improve the accuracy of the model. To get a final prediction. Improving MIMO-DD-3Q Learning model, as shown in Fig 3.

The specific steps to Improving MIMO-DD-3Q Learning model are as follows:

1. Set the training frequency threshold of the model, Dual 3Q Learning initial state model set M, control learning rate *α*, balance future reward decay factor *γ*. In the embodiment of the invention, the state model set M = {LSTM network, Deep-RF network, Transformer network}. Obtain the water quality time sequence data input after filtering in Step 1, and set the sample time sequence length as Nt.
2. Initialize Q*(*S*,*A*), define probability parameter *σ*∈(0,1), and start learning. When *σ*>*ε*, select action *A*_*t*_ randomly with the probability of *σ*. Otherwise, select action *A*_*t*_ = *argmax*Q′ according to Q table, where Q′’ represents the Q value of each predicted action, and select the action with the largest Q value to execute.
3. Carry out the first retraining learning, in the single step time, execute: update the reward value *R*_*ave*_ by Formula (2); Q*(*S*,*A*) is updated by Formula (9); The prediction error is obtained according to the preliminary prediction results of the prediction model.
4. After u times of training, the optimal strategy Q*(*S*,*A*) is obtained after completing the prediction processing of the sample timing series, and the prediction model set, preliminary prediction result set and prediction error set are also obtained.
5. The parameters of the Improving MIMO-DD-3Q Learning model were initialized, and the prediction error set was input into the model for the second training learning. The process of execution in a single time step was described in Step 3.
6. After the second training reaches the threshold, the optimal strategy is obtained, and the prediction model set and the corrected error set are output. In a single time step, the corrected error is compensated to the preliminary prediction result to obtain the predicted index values within the time step.

The Improving MIMO-DD-3Q Learning model parameters stored in the two-training learning will be used to predict water eutrophication. Improving MIMO-DD-3Q Learning model can improve the prediction accuracy of eutrophication data. In order to find the optimal Q learning strategy on the single step prediction, LSTM network, Deep-RF network and Transformer network are used in this step as the state data set for Improving MIMO-DD-3Q Learning model, and each training of Improving MIMO-DD-3Q Learning model is a complete time series prediction.

For MIMO-DD-3Q Learning, the depth model to be predicted at the current moment is selected according to the Q learning strategy in a single step time, and the reward *R*_*ave*_ after the end of the action and the state of the next moment are obtained. At the same time, the preliminary prediction results of the moment, the selected prediction model set at the moment and the error set of the moment are obtained. After the completion of the MIMO-DD-3Q Learning, the model parameters are initialized and the input data is replaced with error sets to obtain a new Q learning strategy. The above operations are repeated in other processes, so as to jointly constitute the prediction process of Improving MIMO-DD-3Q Learning prediction model for lake eutrophication and obtain the final prediction result.

**Fig 4:**
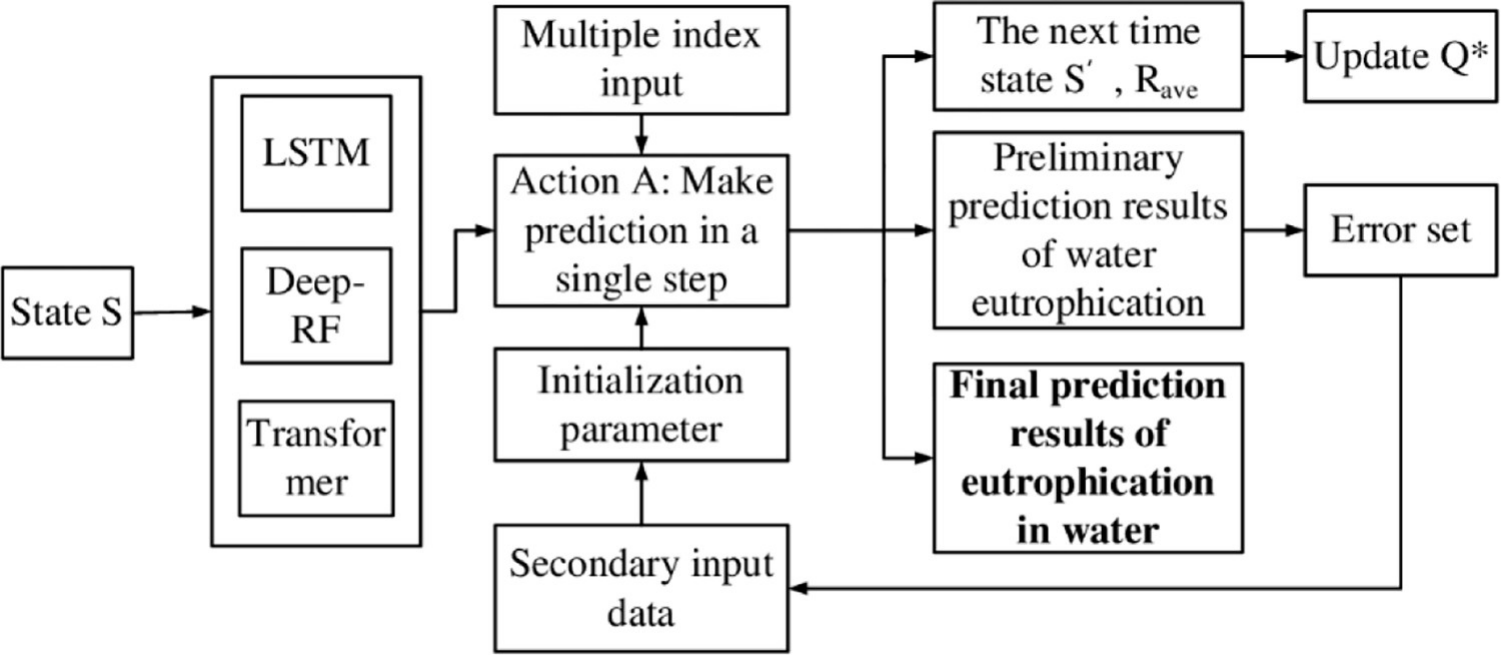
Lake eutrophication prediction flow chart with improving MIMO-DD-3Q Learning.

When the updated Improving MIMO-DD-3Q Learning model is used for real-time lake eutrophication prediction, the water quality time series data currently collected including the eutrophication prediction index are filtered and input into the model. The preliminary prediction result set and prediction error set are obtained through the first prediction, and the corrected error set is obtained through the second prediction. The error is compensated to the preliminary prediction results of corresponding time steps, and the final output of each prediction index value representing lake eutrophication. The complete prediction process is shown in Fig 4.

## 3 Experimental verification

### 3.1 Data set

Taking 12 eutrophication prediction indexes of a river including KMNO_4_, COD,BOD_5_,TOC, NH_3_-N, chroma, conductivity,TDS, turbidity, NO_3_-N, Chl-a and fluoride as examples, the method proposed in this study was used to predict eutrophication of water bodies. The data obtained were screened and processed. The selection time span is from 0 o ’clock on July 26, 2021 to 0 o ’clock on September 5, 2021, during which the sample sampling interval is once every hour, and the time length is 1008 groups of data with 12 data features. In the overall experiment, the first 900 groups of data were selected as the training set of the model, and the first 90 groups of the remaining data were selected as the test set of the model. Specific data are shown in Table 1:

**Table 1:** Experimental data set.

attribute	numerical value	content
Node characteristic	12	KMNO4, COD,BOD5,TOC, NH3-N, chroma, conductivity,TDS, turbidity, NO3-N, Chl-a,fluoride
Time length	From July 26 to September 5	days×24num /day = 1008 Time node
Data partition	(900,90)	The first 900 training sets,The last 90 test sets

### 3.2 RAF and PCA

#### 3.2.1 Recursive average filtering

The water quality data of the area to be studied were collected and the concentration values of various factors were measured. The embodiment of this study included 12 factors such as KMNO_4_, COD,BOD_5_,TOC, NH_3_-N, chroma, conductivity,TDS, turbidity, NO_3_-N, Chl-a and fluoride. The prediction index values of the water to be studied were measured at different time points to obtain the water quality time series data. Each measurement sample contains the concentration values of these factors measured at a point in time.

Lake eutrophication is a phenomenon produced by the joint action of many factors, and the multi-factor data will be affected by certain noise during measurement, and the noise of the data is similar to the Gaussian noise distribution. Since there is noise in the data itself, which affects the prediction effect of the prediction model, the data is smoothed first and processed by means of recursive average. Two sets of sequences a and b are constructed first, which are shown as follows:

a=(1/l,1/l,⋯,1/l) (10)

$$b=(N_{1},N_{2},\cdots ,N_{n})$$ (11)

Where, *l* represents the length of sequence a. The larger the value of *l* is, the smoother the data will be. *n* is the number of samples, and *N*_*n*_ is the n-th sample.

Convolving the two sets of sequences a and b gives the smoothed sequence *b*′, which is expressed as follows:

$$b^{\prime }=[N_{1}*\frac{1}{l},(N_{1},+N_{2})*\frac{1}{l},\cdots ,(N_{1},+N_{2}+\cdots +N_{l})*\frac{1}{l},(N_{2},+N_{3}+\cdots +N_{l+1})*\frac{1}{l},\cdots ,$$

$$\left(N_{n-l},+N_{n-l+1}+\cdots +N_{n})*\frac{1}{l}\right]$$ (12)

**Fig 5:**
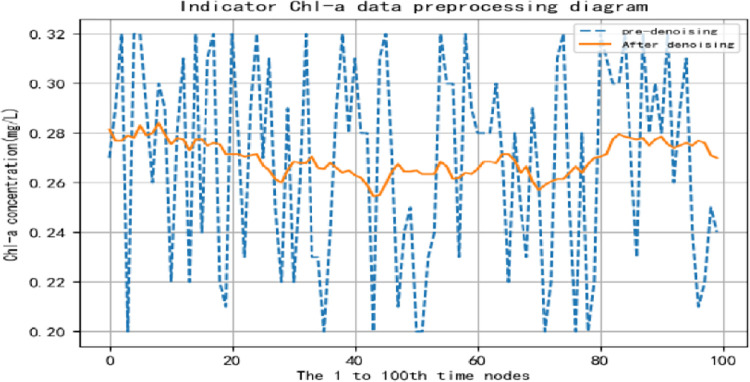
Comparison before and after the removal of Chl-a data noise.

**Fig 6:**
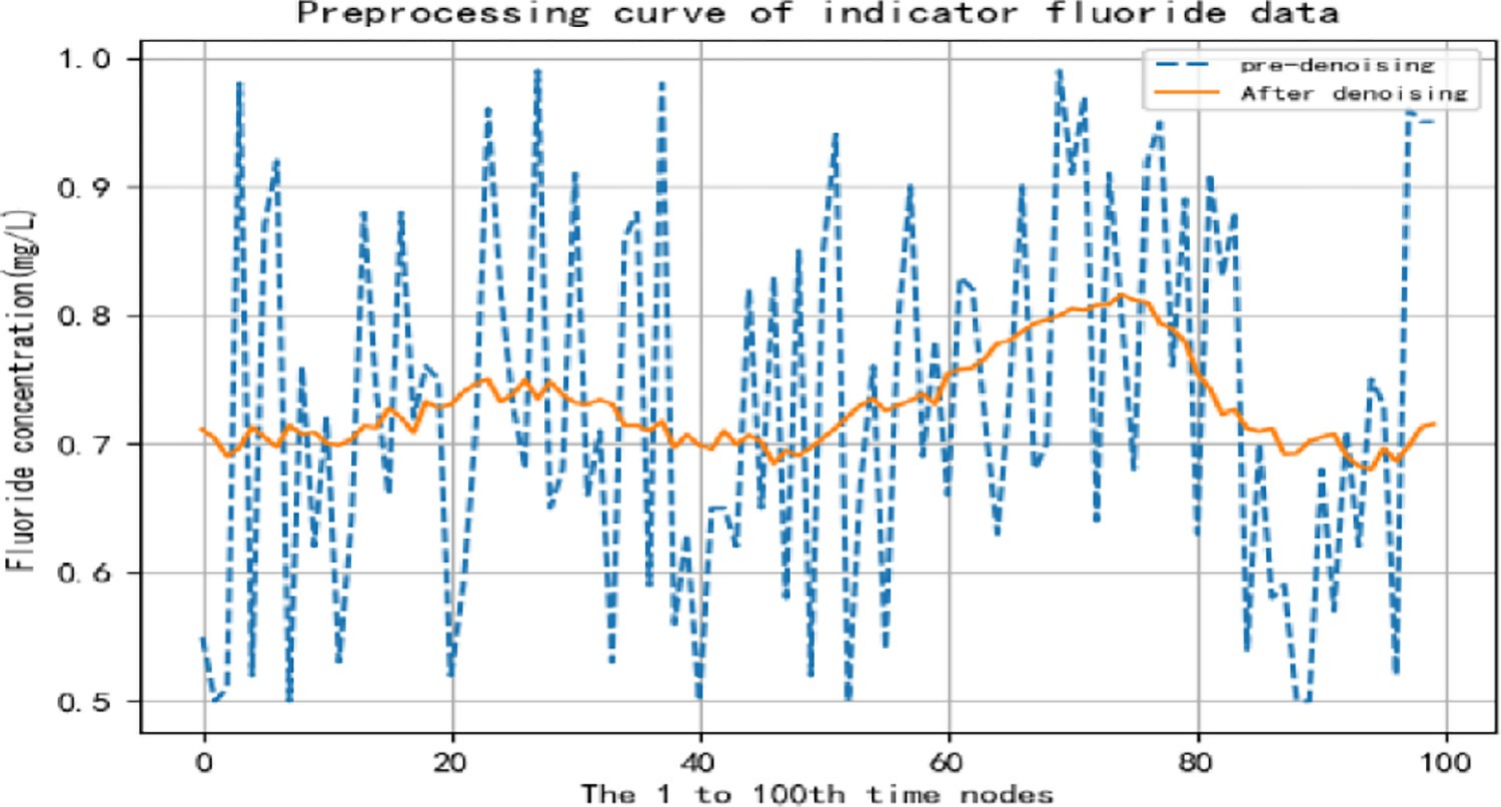
Comparison of fluoride data before and after noise removal.

The smoothed data sample is obtained, and the data comparison before and after de-noising for some factors is shown in Figs 5 and 6.

#### 3.2.2 Principal component analysis

For data with multiple indexes and multidimensional dimensions, principal component analysis can not only reduce and simplify the data, but also judge the effective correlation between various indexes. After the smooth data is obtained by the above method, the initial data matrix is first established with the data samples and the selected prediction indicators of water eutrophication, and the initial data matrix *B* is defined. There are *s* horizontal prediction indicators of water eutrophication and *v* longitudinal data samples. The matrix is shown as follows:

$$B=(\begin{array}{cccc} x_{11} & x_{12} & \cdots & x_{1s}\\ x_{21} & x_{22} & \cdots & x_{2s}\\ \cdots & \cdots & \cdots & \cdots \\ x_{v1} & x_{v2} & \cdots & x_{vs} \end{array})$$ (13)

The data matrix *B* get matrix $\bar{B}$ standardizing, matrix $\bar{B}$ elements in ${\bar{x}}_{jk}$ computation formula is as follows:

$${\bar{x}}_{jk}=\frac{x_{jk}}{\sqrt{\sum_{j=1}^{v}{x_{jk}}^{2}}}$$ (14)

Inspection by KMO and Bartlett again about the suitability of the data matrix $\bar{B}$ principal component analysis, the KMO value is greater than 0.5 to meet the criteria of principal component analysis. If yes, the principal component will be extracted next, and the final number of principal components will be determined by the size of eigenvalue close to one and the total contribution rate of eigenvalue is greater than 85%. According to the standardization of matrix $\bar{B}$ calculation s a predictor of characteristic value, characteristic value contribution rate computation formula is as follows:

$$e_{k}=\frac{\lambda_{k}}{\sum_{k=1}^{s}\lambda_{k}}$$ (15)

$$E_{p}=\sum_{k=1}^{p}e_{k}$$ (16)

Where *e*_*k*_ is the variance interpretation rate of the *k* eigenvalue *λ*_*k*_, and *E*_*p*_ is the sum of the variance interpretation rates of *p* eigenvalues. The number of principal components can be determined according to the obtained results. The component matrix table is obtained by SPSS (Social Science Statistical Software Package). The more approximate the absolute value of the load coefficient of each index in different principal components is 1, the higher the explanation rate of the index to the principal component is. Through all the above processes, the principal component calculation formula is finally determined as follows:

$$F_{i}=m_{i1}X_{1}+m_{i2}X_{2}+,\cdots ,m_{is}X_{s},i=1,2,\cdots ,p$$ (17)

In the formula, *F*_*i*_ represents the *i* principal component, and there are *s* prediction indexes of water eutrophication in total. *X*_*k*_ is the *k* prediction index, and a total of *p* principal components are determined. *m*_*ik*_ is the load coefficient value of the *k* prediction index in the ith principal component.

According to the principal component analysis, it can be proved that all factors are related in the prediction of water eutrophication, but the correlation is strong or weak. The prediction index with high interpretation rate among *p* principal components can be selected according to the actual situation. In this study, 12 predictors were selected for embodiments.

The initial data matrix was constructed according to the data sample and the eutrophication prediction index of water body. After standardization processing, KMO value and Bartlett value were obtained to prove that the data sample was suitable for principal component analysis. The results are shown in Table 2:

**Table 2:** Principal component analysis table.

KMO		0.812
Bartlett sphericity test	Approximate chi-square	151021.87
	df	66
	P	0.000***

Note

***,**, * represent the significance level of 1%, 5% and 10% respectively.

The eigenvalue and contribution rate of eigenvalue were calculated to determine the number of final principal components. The results are shown in Table 3:

**Table 3:** Interpretation of data population variance.

Composition	*λ*_*k*_	VIR (%)	SVIR (%)
1	5.337	44.478	44.478
2	1.626	13.547	58.025
3	1.339	11.156	69.181
4	1.238	10.315	79.496
5	0.886	6.281	85.777
6	0.656	5.466	91.243
7	0.575	4.796	96.039
8	0.277	2.306	98.345
9	0.136	1.133	99.478
10	0.059	0.489	99.967
11	0.003	0.021	99.988
12	0.001	0.012	100

It can be determined from Table 3 that the number of principal components is 5. According to the obtained factor loading coefficient, the ratio coefficients of different indicators in each principal component were analyzed, and the importance of the index was determined according to the common degree (common variance factor), as shown in Table 4 below.

**Table 4:** Factor load coefficient table.

	PC Ⅰ	PC Ⅱ	PC Ⅲ	PC Ⅳ	PC Ⅴ	CD
KMNO4	-0.285	-0.479	0.235	0.596	-0.385	0.87
COD	-0.07	0.018	0.786	-0.165	0.381	0.795
BOD5	0.854	0.396	-0.032	-0.146	0.1	0.918
TOC	0.972	0.147	0.109	0.012	-0.049	0.981
NH3-N	0.211	-0.134	0.182	0.73	0.466	0.846
Chroma	0.97	0.114	0.121	0.038	-0.053	0.972
Conductivity	-0.488	0.737	0.006	0.288	-0.097	0.874
TDS	-0.405	0.782	0.039	0.333	-0.088	0.896
Turbidity	0.967	0.113	0.151	0.049	-0.093	0.982
NO3-N	0.267	-0.078	-0.705	0.226	0.379	0.769
Chl-a	0.708	-0.048	-0.249	0.164	-0.143	0.613
Fluoride	0.827	-0.094	0.151	0.16	-0.194	0.779

### 3.3 Model evaluation index

MAE, RMSE, MAPE were used to predict the model results.

Define the predicted value as: $\hat{Q}=\{{\hat{q}}_{1},{\hat{q}}_{2},\cdots ,{\hat{q}}_{n}\}$, The true value is: ***Q*** = {*q*_1_, *q*_2_,⋯,*q*_*n*_}.

MAE(Mean Absolute Error) is calculated as follows:

$$MAE=\frac{1}{n}\sum_{i=1}^{n}\left|{\hat{q}}_{i}-q_{i}\right|$$

RMSE(Root Mean Square Error) is calculated as follows:

$$RMSE=\sqrt{\frac{1}{n}\sum_{i=1}^{n}{\left({\hat{q}}_{i}-q_{i}\right)}^{2}}$$

MAPE(Mean Absolute Percentage Error) is calculated as follows:

$$MAPE=\frac{1}{n}\sum_{i=1}^{n}|\frac{{\hat{q}}_{i}-q_{i}}{q_{i}}|\times 100\%$$

## 4 Result and discussion

### 4.1 Performance comparison of MIMO-DD-3Q Learning

Firstly, the nonlinear greed factor is introduced to increase the "Exploration" process of Agent in the early stage of Q Learning, so as to quickly update the Q table. With the gradual increase of the number of iterations, the curve of the greed factor gradually becomes flat and gradually approaches a certain value. At this time, the Agent can realize the "Utilization" process in Q learning to the maximum extent. The training times of introducing nonlinear greed factor and fixed greed factor were selected to compare with the updating convergence time of Q table, as shown in Table 5 below:

**Table 5:** Update convergence time of some training times.

Training times	Fixe *ε* ct (s)	Improved *ε* ct (s)
50	11.70	9.77
100	23.71	19.51
150	34.60	28.87
250	58.08	48.15
400	91.83	76.31
700	160.15	133.65
1000	228.09	191.62
1400	320.44	266.47
1750	397.67	334.31
2000	475.77	383.54
2500	571.89	491.14
3000	683.61	581.46

First, the Agent selects the action according to the Q table or selects the action randomly through the probability of greed factor *ε*. The reward value obtained after prediction and the three estimated Q values at the next moment to obtain the three actual Q values at the moment, and then obtains the weight parameter of the final Q value by calculating the average value and the minimum value, and finally updates the final Q value. Q obtained by different iterations of timing data and partially connected moments adopted in this paper is shown in Table 6 below:

**Table 6:** Q table of 3Q Learning with the introduction of weight parameters (part).

Times	t	t+1	t+2	t+3	t+4	t+5
50	-0.069595936	-0.06929062	-0.069868088	-0.069431648	-0.06913566	-0.0691436
100	-0.08068446	-0.080387518	-0.080960999	-0.080534673	-0.09145635	-0.0724022
150	-0.080675865	-0.08040529	-0.080998318	-0.080598831	-0.08033650	-0.0803181
200	-0.084300081	-0.084036831	-0.084631882	-0.097565076	-0.07511443	-0.0846655

### 4.2 Comparison of prediction results of lake eutrophication

First of all, LSTM network, Deep-RF network and Transformer network are selected as the prediction model for Agent action selection, namely the initial state set. Then, the improved parameters are initialized and Q table is initialized, so that Agent can start to learn according to the given "Exploration-Utilization" rule. Then update the Q value of the current moment according to the obtained reward factor and the estimated value of the next moment. With the gradual increase of the number of iterations, the optimal learning strategy is obtained. The selection action is carried out according to the current optimal strategy, and the prediction result of the One-Dual 3Q Learning is obtained. Make the Agent learn again and find the optimal learning strategy, so as to get the final prediction result. Take August 1 from 00:00 to 12:00 as an example. The prediction model selected by the model is shown in Table 7 below:

**Table 7:** Results of 3Q Learning selection model in different time periods.

Time	3Q Learning prediction model	DD-3Q Learning prediction model
0:00~1:00	Deep-RF	Deep-RF
1:00~2:00	LSTM	LSTM
2:00~3:00	LSTM	Deep-RF
3:00~4:00	Deep-RF	Deep-RF
4:00~5:00	Transformer	LSTM
5:00~6:00	Deep-RF	Deep-RF
6:00~7:00	Transformer	Deep-RF
7:00~8:00	LSTM	Deep-RF
8:00~9:00	Deep-RF	LSTM
9:00~10:00	Deep-RF	Transformer
10:00~11:00	Transformer	Transformer
11:00~12:00	Deep-RF	Deep-RF

**Fig 7:**
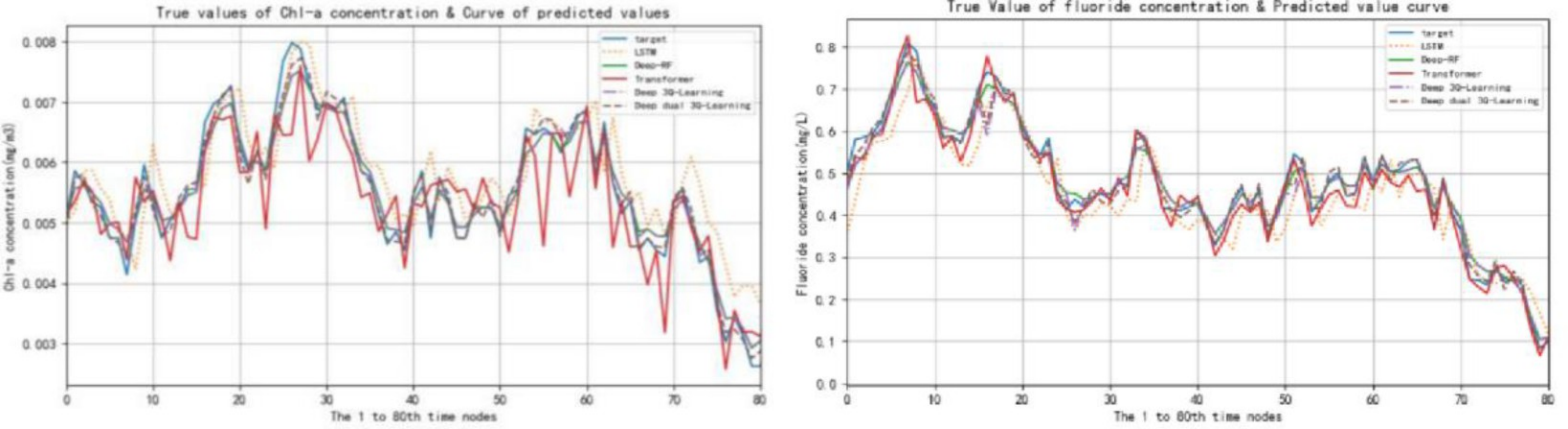
Curves of true and predicted values of five models of Chl-a concentration and fluoride concentration.

**Fig 8:**
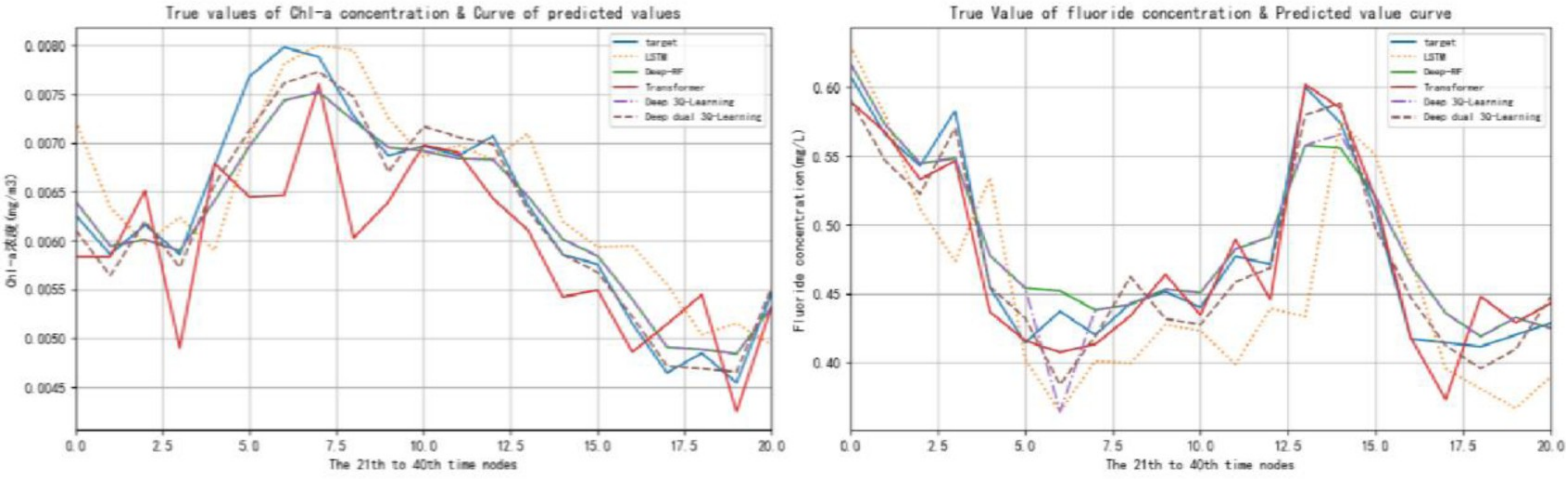
Curve of Chl-a concentration and fluoride concentration at time nodes 21–40.

The lake eutrophication index is taken as the input of model selection action prediction, and the reward factor is calculated according to the obtained results, so as to update the model parameters, make it find the optimal strategy, and then obtain the final prediction result through the optimal strategy. Compare the predicted value of lake eutrophication with the true value curves of LSTM, Deep-RF, Transformer, 3Q Learning model and DD-3Q learning model, and the results are shown in Fig 7. The predicted value and true value curves of each model from time node 21 to time node 40 are shown in Fig 8. The evaluation indicators of each model are shown in Table 8 below:

**Table 8:** Model evaluation indicators.

	MAE	RMSE	MAPE
LSTM	4.97*10^−2^	5.27*10^−2^	0.051%
Deep-RF	3.87*10^−2^	3.37*10–2	0.039%
Transformer	4.30*10^−2^	4.63*10–2	0.046%
3Q Learning	2.90*10^−2^	2.39*10^−2^	0.036%
DD-3Q Learning	1.24*10^−2^	1.52*10^−2^	0.024%

As shown in Fig 8, target represents the target curve, which is represented by DD-3Q Learning in this research method. As can be seen from the figure, Compared with the prediction curves of LSTM model, Deep-RF model, Transformer model and 3Q Learning model, the prediction curves of Chl-a concentration prediction and fluoride concentration prediction based on this research method are closer to the real target curves of Chl-a concentration prediction and fluoride concentration concentration. It can be seen from the evaluation indicators in Table 8. The error between the predicted value and the real value is the smallest and the accuracy is the highest.

### 4.3 Discussion

The purpose of this experiment is that Improving MIMO-DD-3Q Learning model proposed in this study is significantly better than LSTM, Deep-RF, Transformer and One Dual 3Q Learning models in predicting lake eutrophication. Meanwhile, the efficiency of Q learning training is improved by improving Q Learning algorithm. Taking the prediction results of Chl-a concentration index and fluoride index as an example, based on the test results and the curves of the predicted and true values of each model after local amplification, the results of the three error results of each model in Fig 8 and Table 8 can be obtained. Firstly, it can be observed that only using LSTM model for water eutrophication prediction results in the largest error; secondly, only using Transformer model for lake eutrophication prediction results slightly decrease compared with LSTM model; however, Transformer model has greater errors in some data mutation moments. In the single prediction model, the prediction error of Deep-RF model is smaller than the previous two models. In the prediction of lake eutrophication of the One Dual 3Q Learning model, the error is significantly decreased compared with the previous three models. Finally, the prediction error of Improving MIMO-DD-3Q Learning model proposed in this study is the lowest and has an obvious downward trend.

## 5 Conclusion

This paper takes multi-factor water quality data that may cause lake eutrophication as the research object, analyzes the influence of each index on the water eutrophication phenomenon, improves the existing reinforcement learning algorithm, and proposes a lake eutrophication prediction method based on Improving MIMO-DD-3Q Learning model. The following conclusions are obtained through the example verification of the water quality monitoring data of Yongding River in Beijing.

1. For the prediction of lake eutrophication, it is necessary and difficult to accurately predict the data with strong volatility. The traditional single depth prediction model has advantages and disadvantages in predicting the steep and gentle areas of data, and the introduction of Q Learning can combine the advantages of multiple prediction models. At the same time, by taking advantage of the precise decision-making power of reinforcement learning and considering long-term returns, the unified modeling of multi-factor correlation and multi-model combination of lake eutrophication is realized.
2. Aiming at the problems of slow training efficiency of Q Learning model and easy to fall into local optimization, the Q Learning algorithm is improved, and the greedy factor algorithm with arctangent function is proposed, so that the Agent can fully explore the environment in the early stage. Three Q estimates are introduced to update the Q table, and the final Improving MIMO-DD-3Q Learning model is constructed. To improve the training efficiency of the model and reduce the possibility of the model falling into the local optimal as far as possible.
